# Comparative study of a novel selective urate reabsorption inhibitor “dotinurad” among patient groups with different stages of renal dysfunction

**DOI:** 10.1007/s10157-021-02115-7

**Published:** 2021-07-30

**Authors:** Toshinari Takahashi, Takanobu Beppu, Yuji Hidaka, Tatsuo Hosoya

**Affiliations:** 1 Medical Affairs Department, Mochida Pharmaceutical Co., Ltd, 1-22 Yotsuya, Shinjuku City, Tokyo 160-0004 Japan; 2 Medical Affairs Department, Fuji Yakuhin Co., Ltd, 9F Kanda Square Building, 2-2-1 Kandanishiki-cho, Chiyoda City, Tokyo 101-8189 Japan; 3 Akasaka Central Clinic, 3-21-16 Akasaka, Minato City, Tokyo 107-0052 Japan; 4 Jikei University School of Medicine, 3-25-8 Nishi-Shimbashi, Minato City, Tokyo 105-8461 Japan

**Keywords:** Hyperuricemia, Chronic kidney disease, Selective urate reabsorption inhibitor, URAT1 inhibitor, Dotinurad

## Abstract

**Background:**

Dotinurad is a selective urate reabsorption inhibitor (SURI), which selectively inhibits URAT1 to lower serum uric acid levels in patients with hyperuricemia. Herein, the effects of dotinurad were compared among patient groups with different stages of renal dysfunction.

**Methods:**

Patient data from four clinical trials were pooled and divided into four groups according to the stage of renal dysfunction to compare the effects of dotinurad at different stages. The grouping (stages G1–G3b) was based on the estimated glomerular filtration rate (eGFR) of the patients. In addition, patient data from a long-term study (34 or 58 weeks) were evaluated in the same manner.

**Results:**

In the pooled analysis, the percentage of patients achieving a serum uric acid level of ≤ 6.0 mg/dL was 64.7–100.0% at a dose of 2 or 4 mg. In the long-term analysis, the percentage of patients achieving a serum uric acid level of ≤ 6.0 mg/dL was 60.0–100.0% at a dose of 2 or 4 mg. Although the outcomes in stage G3b were worse due to higher baseline serum uric acid levels, satisfactory outcomes were observed in all stages. Even in stages G3a and G3b, when renal function declined, the eGFR remained constant throughout the dose period.

**Conclusion:**

The efficacy of dotinurad was confirmed in hyperuricemic patients with normal renal function (stage G1) and mild to moderate renal dysfunction (stage G2–G3b). Dotinurad was found to be effective in the treatment of hyperuricemia in patients with mild to moderate renal dysfunction.

## Introduction

Hyperuricemia results in urate deposition diseases, such as urolithiasis and gouty arthritis. In addition, recent studies have shown that hyperuricemia is closely related to lifestyle diseases, such as chronic kidney disease, hypertension, and diabetes mellitus [1–3]. Coupled with the aging of the population and westernization of lifestyle, the number of these lifestyle diseases has been increasing. Because of this trend, it is expected that the number of patients with hyperuricemia will continue to increase in the coming decades [4].

According to the latest Japanese guidelines for the management of hyperuricemia and gout [1], uric acid production inhibitors are recommended for patients with chronic kidney disease (CKD; stage 4 or higher). However, in other patient populations, it was suggested that no difference in outcomes would be expected if either uric acid production inhibitors or uricosuric drugs were selected. Although many Japanese patients with hyperuricemia have the characteristic of underexcretion, uricosuric drugs are not often used to treat such patients [5]. Lesinurad, a selective urate reabsorption inhibitor (SURI), which is a URAT1 inhibitor that does not affect other urate transporters, such as OAT1 and OAT3, has recently been approved in the United States and Europe [6]. Lesinurad is indicated in combination with an XOI for patients with gout who failed to achieve the target serum uric acid level due to serious, acute kidney injury, which was observed with high-dose lesinurad monotherapy in a clinical study [7].

Although “underexcretion type” hyperuricemia is observed in many patients with renal impairment, uricosuric drugs (including lesinurad) have not been used because of safety concerns. Thus, there is a need for safer drugs with sufficient serum uric acid-lowering effects [5]. Dotinurad is a novel SURI for the treatment of hyperuricemia. It was developed to improve the safety problems associated with conventional uricosuric drugs while maintaining a strong serum uric acid-lowering effect. Previous clinical trials have confirmed the non-inferiority of dotinurad to benzbromarone [8] and febuxostat [9]. In addition, the safety and efficacy of long-term use have already been evaluated [10]. In Japan, as mentioned above, the number of hyperuricemic patients with renal impairment is expected to increase due to lifestyle diseases [11]. In this situation, it is essential to evaluate the safety of dotinurad in patients with renal impairment. In this study, the safety and efficacy of dotinurad, including its long-term use, were examined in patients with renal impairment based on the analyses of pooled clinical data from several trials conducted to date.

## Methods

### Data set

Clinical data of patients who were administered dotinurad were extracted from four clinical trials and gathered as a data set. The details are as follows: 60 cases were extracted from the phase IIa trial (NCT02344862) [12], 160 from the phase IIb trial (NCT02416167) [13], 102 from the non-inferiority trial to benzbromarone (NCT03100318) [8], and 99 from the non-inferiority trial to febuxostat (NCT03372200) [9]. For long-term evaluation, 326 cases from a long-term trial (NCT03006445) [10] were used as the data set. The inclusion and exclusion criteria for the study subjects were the same in all trials in which patients with “underexcretion type” hyperuricemia were recruited. In four clinical trials, dotinurad was initiated at a dose of 0.25 mg or 0.5 mg daily and was gradually increased to the maintenance dose (0.5, 1, 2, 4 mg). In a long-term study, dotinurad was initiated at a dose of 0.5 mg daily and increased up to 2 or 4 mg. The treatment periods were 8–14 weeks in the four trials and 34 or 58 weeks in the long-term trials. The details of each trial are summarized in Table 1.

**Table 1:** Clinical trials of dotinurad

Clinical trial gov ID	Study objectives	Dotinurad dose (day)	Dosing period	No. of total patients
NCT02344862	Dose response, optimal dose and safety (phase 2a)	0.25 → 0.5 → 1, 2, 4 mg placebo	8 weeks	80
NCT02416167	Dose response, optimal dose and safety (phase 2b)	0.25 → 0.5 → 0.5, 1, 2, 4 mg placebo	12 weeks	199
NCT03100318	Non-inferiority test to benzbromarone and evaluation of safety	0.5 → 1 → 2 mg benzbromarone 25 → 50 → 50 mg	14 weeks	Dotinurad:102 benzbromarone:98
NCT03372200	Non-inferiority test to febuxostat and evaluation of safety	0.5 → 1 → 2 mg febuxostat 10 → 20 → 40 mg	14 weeks	Dotinurad:99 febuxostat:100
NCT03006445	Long-term efficacy and safety	0.5 → 1 → 2 mg 0.5 → 1 → 2 → 4 mg	34 or 58 weeks	326

In the pooled analysis, 421 cases were divided into four groups according to eGFR, which is a standard indicator of the stage of renal dysfunction. Grouping was as follows: stage G1, eGFR ≥ 90 mL/min/1.73 m^2^; stage G2, 60 mL/min/1.73 m^2^ ≤ eGFR < 90 mL/min/1.73 m^2^; stage G3a, 45 mL/min/1.73 m^2^ ≤ eGFR < 60 mL/min/1.73 m^2^; and stage G3b, 30 mL/min/1.73 m^2^ ≤ eGFR < 45 mL/min/1.73 m^2^. This grouping was performed in reference to the Japanese guidelines for the treatment of CKD [14]. In each stage, patient data were further divided into dose sub-groups (0.5–4 mg daily), and outcomes were examined in each sub-group. In the long-term analysis, 7 out of 326 patients were excluded, because the maintenance dose had not been administered for the prescribed duration, and the other 319 cases were extracted and divided into four groups in the same manner as described above (stages G1–G3b), and were evaluated in the 2 or 4 mg dose sub-group.

### Study endpoints

In the pooled analysis, the percentage change in serum uric acid level from the baseline, and percentage of patients achieving a serum uric acid level of ≤ 6.0 mg/dL at the end of the dose period were examined in stages G1–G3b. In the long-term analysis, the incidence of adverse events (AEs) during the dose period, change in eGFR, percentage change in serum uric acid level from the baseline, and percentage of patients achieving a serum uric acid level of ≤ 6.0 mg/dL at 34 and 58 weeks were evaluated in each group. In the analysis of eGFR, paired *t* tests were conducted to evaluate significant changes between the baseline values and values after the dose period.

### Statistical analysis

Statistical analyses were conducted using JMP10 software (SAS Institute, Cary, NC, USA). Summary statistics and 95% confidence interval (CI) were calculated. In addition, a paired *t* test was conducted in the analysis of eGFR, where the significance level was set at 0.05, in a two-tailed test (*p* < 0.05).

## Results

### Pooled analysis of the four clinical trials

Pooled patient data were divided into four groups (G1–G3b) according to the stage of renal dysfunction. The background information of patients is presented in Table 2. The total number of analyzed cases was 421. There were no differences between the stages, except in age.

**Table 2:** Patient background information of the combined analysis (4 trials)

Item			Subcategory	Pooled analysis				
				Overall	G1	G2	G3a	G3b
Number of cases				421	32	297	76	16
Age (Mean ± S.D.)				55.3 ± 11.0	44.4 ± 10.2	54.3 ± 10.4	61.2 ± 8.6	68.3 ± 6.9
Sex [ratio(%)]			Male	417(99.0)	32(100.0)	295(99.3)	74(97.4)	16(100.0)
			Female	4(1.0)	0(0.0)	2(0.7)	2(2.6)	0(0.0)
Baseline serum uric acid levels (Mean ± S.D.)				8.85 ± 1.13	8.81 ± 1.21	8.79 ± 1.10	8.93 ± 1.17	9.36 ± 1.28
Dosage of dotinurad [ratio (%)]			0.5 mg	39(9.3)	4(12.5)	25(8.4)	8(10.5)	2(12.5)
			1 mg	62(14.7)	7(21.9)	46(15.5)	6(7.9)	3(18.8)
			2 mg	259(61.5)	17(53.1)	179(60.3)	53(69.7)	10(62.5)
			4 mg	61(14.5)	4(12.5)	47(15.8)	9(11.8)	1(6.3)
BMI (Mean ± S.D.)				26.34 ± 4.00	28.56 ± 5.44	26.45 ± 3.88	25.24 ± 3.45	25.07 ± 3.25
Baseline eGFR value (mL/min/1.73 m^2^) (Mean ± S.D.)				69.8 ± 14.1	98.4 ± 9.4	72.4 ± 7.9	53.6 ± 4.3	41.1 ± 3.0
Drinking habit [ratio(%)]			No	144(34.2)	10(31.3)	93(31.3)	32(42.1)	9(56.3)
			Yes	277(65.8)	22(68.8)	204(68.7)	44(57.9)	7(43.8)
Diagnostics [ratio(%)]			Gout or gouty tophus	344(81.7)	28(87.5)	250(84.2)	57(75.0)	9(56.3)
			Hyperuricemia	77(18.3)	4(12.5)	47(15.8)	19(25.0)	7(43.8)
Type of hyperuricemia [ratio(%)]			Underexcretion	364(86.5)	29(90.6)	253(85.2)	67(88.2)	15(93.8)
			Combined or normal	57(13.5)	3(9.4)	44(14.8)	9(11.8)	1(6.3)
Complications [ratio(%)]	Hyperlipidemia		No	203(48.2)	19(59.4)	137(46.1)	40(52.6)	7(43.8)
			Yes	218(51.8)	13(40.6)	160(53.9)	36(47.4)	9(56.3)
	Diabetes Mellitus*		No	397(94.3)	32(100.0)	278(93.6)	72(94.7)	15(93.8)
			Yes	24(5.7)	0(0.0)	19(6.4)	4(5.3)	1(6.3)
	Hypertension		No	199(47.3)	18(56.3)	148(49.8)	31(40.8)	2(12.5)
			Yes	222(52.7)	14(43.8)	149(50.2)	45(59.2)	14(87.5)
Concurrent medicine [ratio(%)]	Antihypertensives	Thiazides	No	385(91.4)	32(100.0)	276(92.9)	67(88.2)	10(62.5)
			Yes	36(8.6)	0(0.0)	21(7.1)	9(11.8)	6(37.5)
		ARBs	No	288(68.4)	26(81.3)	206(69.4)	49(64.5)	7(43.8)
			Yes	133(31.6)	6(18.8)	91(30.6)	27(35.5)	9(56.3)

^*^One case was Type 1. Other cases were Type 2 diabetes

The dose period of dotinurad was 8–14 weeks in the trials. Serum uric acid levels at baseline and after the dose period, the changes from baseline levels, and their ratio (%) were summarized for each stage (Table 3). In each stage, the outcomes were summarized in each dose sub-group (0.5–4 mg) (Table 4).

**Table 3:** Outcomes of the pooled analysis in each stage

Stages	Cases (at the baseline)	Baseline serum uric acid level (mg/dL)	Cases (after the dose period)	Serum uric acid levels after the dose period (mg/dL)	Changes from the baseline (%)	95% CI	Changes from the baseline (mg/dL)	95% CI	Percentage of patients achieving a serum uric acid level of ≤ 6.0 mg/dL (%)
Overall	421	8.85 ± 1.13	420	5.00 ± 1.47	43.40 ± 15.20	41.94–44.85	− 3.85 ± 1.46	− 3.99 to − 3.71	78.3
G1	32	8.88 ± 1.21	32	5.49 ± 1.51	37.18 ± 17.68	31.06–43.31	− 3.39 ± 1.92	− 4.05 to − 2.72	68.8
G2	297	8.79 ± 1.10	296	4.88 ± 1.41	44.52 ± 14.46	42.87–46.17	− 3.92 ± 1.37	− 4.07 to − 3.76	82.4
G3a	76	8.93 ± 1.17	76	5.14 ± 1.67	42.63 ± 16.56	38.91–46.35	− 3.79 ± 1.56	− 4.14 to − 3.44	68.4
G3b	16	9.36 ± 1.28	16	5.65 ± 1.06	38.66 ± 13.85	31.88–45.45	− 3.71 ± 1.59	− 4.49 to − 2.93	68.8

Mean ± S.D

**Table 4:** Outcomes of the pooled analysis in the dose subgroups

Stage	Dose	Cases (at the baseline)	Baseline serum uric acid level (mg/dL)	Cases (after the dose period)	Serum uric acid levels after the dose period (mg/dL)	Changes from the baseline (%)	95% CI	Changes from the baseline (mg/dL)	95% CI	Percentage of patients achieving a serum uric acid level of ≤ 6.0 mg/dL (%)
Overall	0.5 mg	39	9.02 ± 1.20	39	7.04 ± 1.34	21.81 ± 11.35	18.24–25.37	− 1.98 ± 1.11	− 2.33 to − 1.63	23.1
	1 mg	62	8.83 ± 1.04	61	5.75 ± 1.01	34.84 ± 9.59	32.44–37.25	− 3.10 ± 0.98	− 3.34 to − 2.85	68.9
	2 mg	259	8.80 ± 1.12	259	4.90 ± 1.19	44.23 ± 12.09	42.76–45.70	− 3.90 ± 1.23	− 4.05 to − 3.75	84.2
	4 mg	61	8.94 ± 1.23	61	3.40 ± 0.95	62.22 ± 8.48	60.09–64.34	− 5.55 ± 0.95	− 5.79 to − 5.31	98.4
G1	0.5 mg	4	9.23 ± 1.53	4	6.90 ± 0.81	24.62 ± 6.94	17.81–31.42	− 2.33 ± 1.01	− 3.32 to − 1.33	25.0
	1 mg	7	8.49 ± 1.30	7	6.16 ± 1.45	26.86 ± 14.26	16.30–37.42	− 2.33 ± 1.61	− 3.52 to − 1.14	85.7
	2 mg	17	8.63 ± 0.94	17	5.42 ± 1.24	37.03 ± 13.71	30.51–43.55	− 3.21 ± 1.30	− 3.83 to − 2.60	64.7
	4 mg	4	10.30 ± 1.08	4	3.25 ± 0.45	68.47 ± 2.56	65.95–70.98	− 7.05 ± 0.78	− 7.81 to − 6.29	100.0
G2	0.5 mg	25	8.82 ± 1.11	25	6.85 ± 1.35	22.47 ± 9.88	18.60–26.35	− 1.97 ± 0.88	− 2.31 to − 1.62	28.0
	1 mg	46	8.83 ± 0.98	45	5.63 ± 0.94	36.46 ± 8.75	33.90–39.01	− 3.24 ± 0.86	− 3.49 to − 2.99	71.1
	2 mg	179	8.78 ± 1.13	179	4.81 ± 1.14	45.13 ± 11.44	43.45–46.80	− 3.97 ± 1.18	− 4.14 to − 3.80	88.8
	4 mg	47	8.79 ± 1.13	47	3.39 ± 0.98	61.67 ± 9.01	59.09–64.24	− 5.40 ± 0.87	− 5.64 to − 5.15	97.9
G3a	0.5 mg	8	9.54 ± 1.01	8	7.88 ± 1.45	16.86 ± 16.59	5.36–28.35	− 1.66 ± 1.66	− 2.81 to − 0.51	0.0
	1 mg	6	9.03 ± 1.05	6	5.93 ± 1.05	34.70 ± 4.24	31.31–38.09	− 3.10 ± 0.13	− 3.20 to − 3.00	50.0
	2 mg	53	8.83 ± 1.17	53	4.96 ± 1.29	43.86 ± 12.86	40.40–47.32	− 3.88 ± 1.27	− 4.22 to − 3.53	75.5
	4 mg	9	8.92 ± 1.39	9	3.26 ± 0.75	63.57 ± 5.46	60.00–67.14	− 5.67 ± 0.97	− 6.30 to − 5.04	100.0
G3b	0.5 mg	2	9.10 ± 2.83	2	6.40 ± 0.85	27.62 ± 13.17	9.37–45.88	− 2.70 ± 1.98	− 5.44 to − 0.04	50.0
	1 mg	3	9.07 ± 1.68	3	6.30 ± 0.61	29.53 ± 9.80	18.45–40.62	− 2.77 ± 1.39	− 4.34 to − 1.20	33.3
	2 mg	10	9.34 ± 0.93	10	5.33 ± 1.15	42.38 ± 13.86	33.79–50.98	− 4.01 ± 1.52	− 4.95 to − 3.07	80.0
	4 mg	1	11.00 ± –	1	5.40 ± –	50.91 ± –	–	− 5.60 ± –	–	100.0

Mean ± S.D

The outcomes in all stages were as follows. When dotinurad (0.5 mg) was administered, the percentage change in serum uric acid levels from the baseline was 21.81% ± 11.35%, and the percentage of patients achieving serum uric acid levels ≤ 6.0 mg/dL was 23.1%. When 1 mg was administered, these outcomes were 34.84% ± 9.59% and 68.9%, when 2 mg was administered, they were 44.23% ± 12.09% and 84.2%, and when 4 mg was administered, the outcomes were 62.22% ± 8.48% and 98.4%, respectively. As described above, both outcomes improved with the dosage of dotinurad. Dose dependency was found to be the same in each stage, and sufficient outcomes were observed when a dose of 2 mg or higher was administered in all stages. In stages G2–G3b, when the renal function of patients is impaired, the percentage of patients achieving the serum uric acid levels ≤ 6.0 mg/dL was 88.8% in the G2 stage, 75.5% in the G3a stage, and 80.0% in the G3b stage when 2 mg dotinurad was administered. When 4 mg was administered, the outcome was further improved to 97.9% in G2, 100% in G3a, and 100% in G3b. These data indicate the efficacy of dotinurad in patients with mild-to-moderate renal dysfunction.

### Long-term analysis

Patient background information at baseline in stages G1–G3b and the whole population is summarized in Table 5. The number of analyzed cases was 319, which comprised 24 stage G1 patients, 225 stage G2 patients, 61 stage G3a patients, and 9 stage G3b patients. Similar to the results of the pooled analysis, no differences were found between the stages, except in age.

**Table 5:** Patient background information of long-term analysis

Item			Subcategory	Long-term analysis				
				Overall	G1	G2	G3a	G3b
Number of cases				319	24	225	61	9
Age (Mean ± S.D.)				53.8 ± 10.5	48.0 ± 9.9	52.3 ± 10.0	60.3 ± 8.8	64.6 ± 5.2
Sex [ratio(%)]			Male	317(99.4)	24(100.0)	223(99.1)	61(100.0)	9(100.0)
			Female	2(0.6)	0(0.0)	2(0.9)	0(0.0)	0(0.0)
Baseline serum uric acid levels (Mean ± S.D.)				8.78 ± 1.13	8.79 ± 1.07	8.73 ± 1.11	8.77 ± 1.07	10.16 ± 1.44
Dosage of dotinurad [ratio (%)]			0.5 mg	–	–	–	–	–
			1 mg	–	–	–	–	–
			2 mg	276(86.5)	20(83.3)	194(86.2)	57(93.4)	5(55.6)
			4 mg	43(13.5)	4(16.7)	31(13.8)	4(6.6)	4(44.4)
BMI(Mean ± S.D.)				26.44 ± 3.79	26.46 ± 5.15	26.46 ± 3.68	26.14 ± 3.53	27.97 ± 4.48
Baseline eGFR value (mL/min/1.73 m^2^) (Mean ± S.D.)				69.7 ± 13.1	96.0 ± 4.1	72.3 ± 7.7	54.1 ± 3.8	41.1 ± 2.4
Drinking habit [ratio(%)]			No	154(48.3)	4(16.7)	108(48.0)	37(60.7)	5(55.6)
			Yes	165(51.7)	20(83.3)	117(52.0)	24(39.3)	4(44.4)
Diagnostics [ratio(%)]			Gout or gouty tophus	264(82.8)	22(91.7)	188(83.6)	48(78.7)	6(66.7)
			Hyperuricemia	55(17.2)	2(8.3)	37(16.4)	13(21.3)	3(33.3)
Type of hyperuricemia [ratio(%)]			Underexcretion	273(85.6)	20(83.3)	191(84.9)	54(88.5)	8(88.9)
			Combined or normal	46(14.4)	4(16.7)	34(15.1)	7(11.5)	1(11.1)
Complications [ratio(%)]	Hyperlipidemia		No	188(58.9)	13(54.2)	136(60.4)	35(57.4)	4(44.4)
			Yes	131(41.1)	11(45.8)	89(39.6)	26(42.6)	5(55.6)
	Diabetes Mellitus^a^		No	305(95.6)	23(95.8)	219(97.3)	56(91.8)	7(77.8)
			Yes	14(4.4)	1(4.2)	6(2.7)	5(8.2)	2(22.2)
	Hypertension		No	165(51.7)	12(50.0)	129(57.3)	24(39.3)	0(0.0)
			Yes	154(48.3)	12(50.0)	96(42.7)	37(60.7)	9(100.0)
Concurrent medicine [ratio(%)]	Antihypertensives	Thiazides	No	302(94.7)	22(91.7)	218(96.9)	56(91.8)	6(66.7)
			Yes	17(5.3)	2(8.3)	7(3.1)	5(8.2)	3(33.3)
		ARBs	No	244(76.5)	16(66.7)	185(82.2)	40(65.6)	3(33.3)
			Yes	75(23.5)	8(33.3)	40(17.8)	21(34.4)	6(66.7)

^a^All of them were Type 2 diabetes

The patient outcomes obtained from the safety analysis using the safety population (SP), including AEs at each stage, are shown in Table 6. There were no differences in the incidence of AEs (%) among the four stages. A serious adverse reaction (ADR) was stage I gastric cancer observed in stage G1.

**Table 6:** Adverse reactions

Stages	G1		G2		G3a^a^		G3b	
Number of cases	24		225		62		9	
	Number of patients	Incidence (%)	Number of patients	Incidence (%)	Number of patients	Incidence (%)	Number of patients	Incidence (%)
AEs	15	(62.5)	148	(65.8)	40	(64.5)	6	(66.7)
Adverse reactions (ADRs)	4	(16.7)	50	(22.2)	12	(19.4)	1	(11.1)
Serious AEs	1	(4.2)	5	(2.2)	3	(4.8)	0	(0.0)
Serious ADRs	1	(4.2)	0	(0.0)	0	(0.0)	0	(0.0)
AEs leading to death	0	(0.0)	0	(0.0)	0	(0.0)	0	(0.0)
ADRs leading to death	0	(0.0)	0	(0.0)	0	(0.0)	0	(0.0)
AEs leading to discontinuation	0	(0.0)	9	(4.0)	2	(3.2)	0	(0.0)
ADRs leading to discontinuation	0	(0.0)	5	(2.2)	1	(1.6)	0	(0.0)

AEs leading to discontinuation in stage G2: urinary bladder cancer, nephrolithiasis (4 cases), uncontrolled diabetes mellitus, eczema, acute inflammation of gall bladder, diverticulitis. ADRs leading to discontinuation in stage G2: nephrolithiasis (4 cases), eczema. AEs leading to discontinuation in stage G3a: gastrointestinal stomal tumor, nephrolithiasis. ADRs leading to discontinuation in stage G3a: nephrolithiasis

*The number of AEs contained those of ADRs in the same stage

^a^Because this analysis used the Safety Population (SP), the number of stage G3a was 62, which was larger than other tables by 1 case

Serum uric acid levels at baseline and after the dose period, changes from baseline, and percentage changes in each stage are shown in Table 7. The outcomes in the dose of each stage are shown in Table 8.

**Table 7:** Outcomes of the long-term analysis in each stage

Stage	Cases (at the baseline)	Baseline serum uric acid level (mg/dL)	Cases (after the dose period)	Serum uric acid levels after the dose period (mg/dL)	Changes from the baseline (%)	95% CI	Changes from the baseline (mg/dL)	95% CI	Percentage of patients achieving a serum uric acid level of ≤ 6.0 mg/dL (%)
Overall	319	8.78 ± 1.13	319	4.51 ± 1.15	48.51 ± 11.94	47.20–49.82	− 4.27 ± 1.23	− 4.41 to − 4.14	90.6
G1	24	8.79 ± 1.07	24	4.47 ± 1.23	48.56 ± 15.02	42.55–54.56	− 4.32 ± 1.58	− 4.95 to − 3.69	87.5
G2	225	8.73 ± 1.11	225	4.46 ± 1.17	48.87 ± 12.23	47.27–50.46	− 4.28 ± 1.24	− 4.44 to − 4.11	92.0
G3a	61	8.77 ± 1.07	61	4.63 ± 1.05	47.30 ± 9.95	44.80–49.80	− 4.15± 0.99	− 4.39 to − 3.90	90.2
G3b	9	10.16 ± 1.44	9	5.28 ± 1.02	47.75 ± 8.74	42.04–53.46	− 4.88 ± 1.27	− 5.71 to − 4.05	66.7

Mean ± S.D

**Table 8:** Outcomes of the long-term analysis in the dose subgroups

Stages	Dose	Cases (at the baseline)	Baseline serum uric acid level (mg/dL)	Cases (after the dose period)	Serum uric acid levels after the dose period (mg/dL)	Changes from the baseline (%)	95% CI	Changes from the baseline (mg/dL)	95% CI	Percentage of patients achieving a serum uric acid level of ≤ 6.0 mg/dL (%)
Overall	2 mg	276	8.63 ± 1.03	276	4.53 ± 1.16	47.52 ± 11.99	46.11–48.93	− 4.10 ± 1.16	− 4.24 to − 3.97	89.9
	4 mg	43	9.76 ± 1.25	43	4.41 ± 1.09	54.88 ± 9.49	52.04–57.72	− 5.35 ± 1.09	− 5.67 to − 5.02	90.6
G1	2 mg	20	8.61 ± 1.04	20	4.35 ± 1.26	48.81 ± 15.68	41.94–55.68	− 4.26 ± 1.61	− 4.96 to − 3.55	85.0
	4 mg	4	9.70 ± 0.75	4	5.05 ± 0.94	47.29 ± 13.01	34.54–60.04	− 4.65 ± 1.58	− 6.19 to − 3.11	100.0
G2	2 mg	194	8.57 ± 0.99	194	4.48 ± 1.19	47.69 ± 12.34	45.95–49.42	− 4.09 ± 1.16	− 4.25 to − 3.92	90.7
	4 mg	31	9.74 ± 1.32	31	4.27 ± 1.02	56.25 ± 8.47	53.27–59.23	− 5.47 ± 1.05	− 5.84 to − 5.10	100.0
G3a	2 mg	57	8.69 ± 0.99	57	4.63 ± 1.00	46.73 ± 9.64	44.23–49.24	− 4.06 ± 0.96	− 4.31 to − 3.81	91.2
	4 mg	4	9.90 ± 1.68	4	4.55 ± 1.87	55.36 ± 12.24	43.37–67.35	− 5.35 ± 0.58	− 5.92 to − 4.78	75.0
G3b	2 mg	5	10.40 ± 1.77	5	5.70 ± 0.90	44.85 ± 6.66	39.01–50.69	− 4.70 ± 1.26	− 5.80 to − 3.60	60.0
	4 mg	4	9.85 ± 1.07	4	4.75 ± 1.01	51.38 ± 10.62	40.97–61.79	− 5.10 ± 1.45	− 6.52 to − 3.68	75.0

Mean ± S.D

The outcomes of the whole population were as follows: in 276 cases that were administered 2 mg dotinurad for 34 or 58 weeks, the percentage change in serum uric acid level from baseline was 47.52% ± 11.99%, and the percentage of patients achieving a serum uric acid level ≤ 6.0 mg/dL was 89.9%. In 43 cases that were administered 4 mg dotinurad, these outcomes were 54.88% ± 9.49% and 90.6%, respectively.

The outcomes of different renal dysfunction stages (G1–G3b) were as follows: in stage G1, the percentage change in the serum uric acid level from baseline was 48.81% ± 15.68%, and the percentage of patients achieving a serum uric acid level ≤ 6.0 mg/dL was 85.0% when 2 mg dotinurad was administered. When 4 mg was administered, the outcomes were 47.29% ± 13.01% and 100.0%, respectively. In stage G2, the outcomes were 47.69% ± 12.34% and 90.7% in the 2 mg sub-group and 56.25% ± 8.47% and 100.0% in the 4 mg sub-group, respectively. In stage G3a, the outcomes were 46.73% ± 9.64% and 91.2% in the 2 mg sub-group and 55.36% ± 12.24% and 75.0% in the 4 mg sub-group, respectively. In stage G3b, the outcomes were 44.85% ± 6.66% and 60.0% in the 2 mg sub-group and 51.38% ± 10.62% and 75.0% in the 4 mg sub-group, respectively. In stage G3b, the percentage of patients achieving a serum uric acid level of ≤ 6.0 mg/dL was relatively low. However, this can be attributed to the relatively high serum uric acid level at baseline, and large variations due to the small number of cases, as the change in uric acid level from baseline was the same as that observed in other stages.

The eGFR values at baseline and after the dose period are shown in Table 9 and Appendix Table 10.

**Table 9:** eGFR values during the dose period

Stages	*n*	Baseline eGFR values (mL/min/1.73 m^2^)	*n*	eGFR values after 2 weeks (mL/min/1.73 m^2^)	*n*	eGFR values after 6 weeks (mL/min/1.73 m^2^)	*n*	eGFR values after 18 weeks (mL/min/1.73 m^2^)	*n*	eGFR values after 34 weeks (mL/min/1.73 m^2^)	Changes	*p* values	*n*	eGFR values after 58 weeks (mL/min/1.73 m^2^)	Changes	*p* values
Overall	319	69.7 ± 13.1	318	68.9 ± 13.8	319	69.2 ± 13.6	312	68.5 ± 14.3	299	70.1 ± 13.8	− 0.6 ± 6.2	0.122	105	69.0 ± 14.2	− 1.1 ± 7.8	0.158
G1	24	96.0 ± 4.1	24	95.6 ± 8.1	24	94.7 ± 6.5	24	95.4 ± 8.8	22	93.6 ± 8.2	− 2.6 ± 8.2	0.146	9	92.1 ± 12.1	− 3.8 ± 11.6	0.359
G2	225	72.3 ± 7.7	224	71.3 ± 8.9	225	71.5 ± 9.0	220	70.9 ± 9.4	211	73.0 ± 9.4	0.8 ± 6.1	0.049*	77	70.4 ± 10.5	− 0.8 ± 7.9	0.349
G3a	61	54.1 ± 3.8	61	54.0 ± 5.6	61	55.0 ± 6.5	59	53.3 ± 6.5	57	55.2 ± 6.2	0.2 ± 2.8	0.209	17	53.4 ± 6.1	− 0.2 ± 2.6	0.576
G3b	9	41.1 ± 2.4	9	40.2 ± 3.4	9	39.6 ± 3.6	9	38.9 ± 5.2	9	40.7 ± 4.7	0.0 ± 1.0	0.794	2	40.0 ± 1.4	0.0 ± 0.5	–

Mean ± S.D

*n* number of patients

^*^p < 0.05(vs baseline)

The dose period for this analysis was 34 or 58 weeks. A total of 299 patients were examined after 34 weeks, and 105 patients who participated early period in the study were observed to have completed 58 weeks of intervention. The baseline eGFR value in stage G1 was 96.0 ± 4.1 mL/min/1.73 m^2^ and 93.6 ± 8.2 mL/min/1.73 m^2^ after 34 weeks, 93.1 ± 12.2 mL/min/1.73 m^2^ after 58 weeks. In stage G2, the baseline values were 72.3 ± 7.7 mL/min/1.73 m^2^, 73.0 ± 9.4 mL/min/1.73 m^2^ after 34 weeks, and 70.4 ± 10.5 mL/min/1.73 m^2^ after 58 weeks. In stage G3a, the baseline values were 54.1 ± 3.8 mL/min/1.73 m^2^, 55.2 ± 6.2 mL/min/1.73 m^2^ after 34 weeks, 53.4 ± 6.1 mL/min/1.73 m^2^ after 58 weeks. In stage G3b, the baseline values were 41.1 ± 2.4 mL/min/1.73 m^2^, 40.7 ± 4.7 mL/min/1.73 m^2^ after 34 weeks, and 40.0 ± 1.4 mL/min/1.73 m^2^ after 58 weeks. The results of the paired *t* test (*p* values are shown in Table 9), a significant change was observed in stage G2 after 34 weeks of treatment (*p* = 0.049). However, no significant change was observed after 58 weeks in the same stage or in other stages between the baseline values and values after the dose period. In stages G3a and G3b, when the renal function of the patients is impaired, the eGFR values remained unchanged throughout the dose period (Appendix Table 10).

## Discussion

Changes in serum uric acid levels from baseline in stages G1–G3b are summarized in Tables 3 and 7. When dotinurad was administered for 8–14 weeks, changes in serum uric acid levels from baseline were similar between the four stages. Even in stages G3a and G3b, the efficacy of dotinurad was similar to that of the other stages. Based on this result, it was confirmed that dotinurad monotherapy is expected to have sufficient uric acid-lowering effects in patients with impaired renal function. As the serum uric acid level-lowering effect of dotinurad is dose dependent, the serum uric acid levels after the dose period observed in the pooled analysis (including patient groups administered 0.5 or 1 mg dotinurad) were higher than those observed in the long-term analysis.

In the long-term analysis, in which 2 or 4 mg dotinurad was administered during the maintenance period, the changes in the serum uric acid levels from baseline were similar in all stages. In addition, the percentage of patients achieving a serum uric acid level of ≤ 6.0 mg/dL was approximately 90% in stages G1, G2, and G3a. In these stages, dotinurad is expected to have a satisfactory effect. In stage G3b, although the changes in the serum uric acid level from baseline were higher than those in the other stages, the percentage of patients achieving a serum uric acid level of ≤ 6.0 mg/dL was only approximately 67%. This was thought to be due to the relatively higher baseline serum uric acid level in this stage. With regard to safety in long-term use, the risks of AEs were similar in all stages, and no particular risk was observed in patient groups with an impaired renal function. Although a significant change in the eGFR value was observed after 34 weeks in stage G2, it was difficult to identify the clinical importance of this change, because no significant change was observed after 58 weeks in the same group. Overall, the eGFR was stable during the dose period in all stages, and no aggravation was observed (Table 9; Appendix Table 10).

Dotinurad monotherapy showed a satisfactory effect in the treatment of patients with hyperuricemia whose eGFR values were 30 mL/min/1.73 m^2^ or higher, and efficacy and safety were similar in different stages of renal dysfunction. In addition, a decline in the eGFR was not observed during the dose period. From the perspective of the eGFR, there was no increase in the renal load during treatment with dotinurad.

On the contrary, in a study using lesinurad, an SURI that has been approved in the USA and EU, elevations in the serum creatinine levels and an increased risk of renal dysfunction were observed in patients receiving high-dose treatments (400 mg) [7]. Further research, focusing on mechanism and dose dependency, is essential to clarify the relationship between SURI and renal load. As this study was a pooled analysis of multiple trials, there was a large variation in the number of cases in the study groups. Therefore, heterogeneity bias cannot be excluded, and the statistical reliability is limited. The outcomes of stage G3b should be evaluated carefully, because the sample size was small in this group. To gain a better understanding, prospective clinical intervention studies in patients with CKD are desirable in the future.

## Conclusions

In Japan, the number of patients with renal impairment is expected to increase due to the growing incidence of lifestyle-related diseases. This study proved that dotinurad could be an effective treatment option for hyperuricemia in such populations.

## Appendix

See Table 10.

**Table 10:** eGFR values during the dose period

	Run-in period		2 weeks		6 weeks		10 weeks		14 weeks		18 weeks		22 weeks		26 weeks	
	*n*	Mean ± S.D	*n*	Mean ± S.D	*n*	Mean ± S.D	*n*	Mean ± S.D	*n*	Mean ± S.D	*n*	Mean ± S.D	*n*	Mean ± S.D	*n*	Mean ± S.D
Overall	319	69.7 ± 13.1	318	68.9 ± 13.8	319	69.2 ± 13.6	314	69.3 ± 13.4	312	68.9 ± 14.3	312	68.5 ± 14.3	307	68.7 ± 14.0	305	69.1 ± 14.4
G1	24	96.0 ± 4.1	24	95.6 ± 8.1	24	94.7 ± 6.5	24	92.4 ± 7.5	24	95.8 ± 7.2	24	95.4 ± 8.8	22	93.0 ± 9.1	22	96.5 ± 9.1
G2	225	72.3 ± 7.7	224	71.3 ± 8.9	225	71.5 ± 9.0	222	71.8 ± 9.1	219	71.4 ± 9.5	220	70.9 ± 9.4	216	71.5 ± 9.4	216	71.5 ± 9.6
G3a	61	54.1 ± 3.8	61	54.0 ± 5.6	61	55.0 ± 6.5	59	55.0 ± 6.2	60	53.5 ± 6.2	59	53.3 ± 6.5	60	54.3 ± 7.9	58	53.9 ± 6.9
G3b	9	41.1 ± 2.4	9	40.2 ± 3.4	9	39.6 ± 3.6	9	39.0 ± 2.9	9	40.3 ± 3.1	9	38.9 ± 5.2	9	38.9 ± 4.2	9	40.8 ± 3.8

	30 weeks		34 weeks		38 weeks		42 weeks		46 weeks		50 weeks		54 weeks		58 weeks	
	*n*	Mean ± S.D	*n*	Mean ± S.D	*n*	Mean ± S.D	*n*	Mean ± S.D	*n*	Mean ± S.D	*n*	Mean ± S.D	*n*	Mean ± S.D	*n*	Mean ± S.D
Overall	302	69.9 ± 14.1	299	70.1 ± 13.8	107	70.8 ± 13.8	106	69.8 ± 13.8	105	69.9 ± 15.3	105	68.9 ± 14.5	104	69.5 ± 14.3	105	69.0 ± 14.2
G1	22	95.0 ± 7.8	22	93.6 ± 8.2	10	93.0 ± 9.6	10	94.4 ± 8.8	10	99.2 ± 7.8	9	97.1 ± 9.0	9	97.9 ± 6.7	9	92.1 ± 12.1
G2	213	72.6 ± 9.5	211	73.0 ± 9.4	78	72.3 ± 9.8	77	71.0 ± 9.2	76	70.9 ± 9.6	77	70.1 ± 9.5	76	70.6 ± 9.2	77	70.4 ± 10.5
G3a	58	55.2 ± 7.2	57	55.2 ± 6.2	17	54.6 ± 6.0	17	53.9 ± 6.2	17	52.2 ± 7.4	17	52.2 ± 5.7	17	53.0 ± 6.0	17	53.4 ± 6.1
G3b	9	40.1 ± 4.2	9	40.7 ± 4.7	2	40.5 ± 2.1	2	39.0 ± 1.4	2	38.5 ± 0.7	2	38.0 ± 1.4	2	38.5 ± 0.7	2	40.0 ± 1.4

*n* number of patients
